# Health outcomes of lifestyle treatment for patients with obesity before and during the COVID-19 pandemic—a retrospective pre-post study

**DOI:** 10.3389/fpubh.2025.1649655

**Published:** 2025-07-28

**Authors:** Sadegh Alizadeh, Sophie Grindheim, Christian A. Klöckner, Ingrid S. Følling

**Affiliations:** ^1^Department of Neuromedicine and Movement Science, Faculty of Medicine and Health Science, Norwegian University of Science and Technology, Trondheim, Norway; ^2^Department of Clinical and Molecular Medicine, Faculty of Medicine and Health Sciences, Norwegian University of Science and Technology, Trondheim, Norway; ^3^Centre for Obesity Research (ObeCe), Clinic of Surgery, St. Olavs University Hospital, Trondheim, Norway; ^4^Department of Psychology, Norwegian University of Science and Technology, Trondheim, Norway

**Keywords:** obesity, lifestyle treatment, rehabilitation center, COVID-19 pandemic, body mass index, health-related quality of life, physical activity

## Abstract

**Background:**

In Norway, one treatment option for patients with obesity has been a multi-component lifestyle program with a continuous stay at rehabilitation centers. During the COVID-19 pandemic, these stays were reduced, and digital follow-up for patients from home was introduced. How these changes affected patients’ health outcomes after treatment is unknown. This study aimed to investigate health outcomes for patients who attended lifestyle treatment and to compare patients who attended treatment before COVID-19 with patients who attended during COVID-19.

**Method:**

A retrospective pre-post design was used. A total of 103 patients (mean age 45.4 years, 64% women) with a mean Body Mass Index (BMI) of 42.3 kg/m^2^ were included of whom 53 attended treatment before COVID-19 and 50 attended treatment during COVID-19. Health outcomes measured were BMI, Physical Activity (PA) levels, and Health-Related Quality of Life (HRQoL). Data were collected at baseline and after 9 weeks of treatment for both groups.

**Results:**

All patients had a significant reduction in BMI after treatments, with no differences between the groups. PA levels and HRQoL increased for both groups, however, the group that attended treatment before COVID-19 had significantly higher PA levels and HRQoL (*p* < 0.001) than the group that attended during COVID-19.

**Conclusion:**

This study demonstrates that structured lifestyle treatment supports improvements in BMI, HRQoL, and PA levels, even with reduced in-person contact. Although differences were observed between treatment periods, further research is needed to understand how the delivery mode and specific components of digital and in-person treatments affect health outcomes, providing insights to optimize future lifestyle interventions for individuals with obesity.

## Introduction

Obesity is an escalating global public health concern (1). It is defined as a chronic, progressive, relapsing disease (2), and is often associated with several co-morbidities such as type 2 diabetes, cardiovascular diseases, musculoskeletal disorders, chronic kidney disease, neoplasms (3) and psychosocial challenges including stress, depression, mood and anxiety disorders (4). People with obesity are prone to mood and anxiety problems compared to the general population (5). Higher body mass index (BMI) consistently correlates with reduced Health-Related Quality of Life (HRQoL) (6). Defined as a multidimensional concept, HRQoL reflects an individual’s perception of how their physical, psychological, and social functioning are affected by illness and its treatment (7). Notably, people with obesity appear to face more physical than mental challenges, with difficulties in everyday tasks such as work, studies, family responsibilities, or leisure activities (8), which contributes to their reduced quality of life (9).

Sustaining weight loss and maintaining mental health were challenging for those with obesity during periods of restriction and isolation during the COVID-19 pandemic (10). While restrictions significantly reduced Physical Activity (PA), increased sedentary time (11, 12), shifted toward lower-intensity activities (13), and mental health including heightened depression, anxiety, and stress, along with a diminished capacity to manage stress (12), there were disproportionate increased effects on people with obesity compared to the general population (11–13). Even in the absence of COVID-19, it is well-known that decreased PA is linked to reduced mental well-being and quality of life, particularly in those with severe obesity (14).

While the pandemic prompted major adjustments in healthcare delivery (15) and enabled patients with obesity to continue receiving dietary counseling, psychological support, and medical advice via online appointments (16), some digital weight loss programs had limited success. For example, one study reported that over 50% of patients with obesity who attended a weight management program during COVID-19 had reduced PA leading to a 61% increase in weight gain and a decline in mental health (15). Another study of a digital weight loss program found that 77% of individuals experienced increased stress due to more mental health challenges as well as more difficulties finding time for weight loss efforts (17).

This current study concerns lifestyle treatment before and during COVID-19 pandemic in Norway. For background information, Norwegians with a BMI of ≥ 35 kg/m^2^ and co-morbidities, or a BMI ≥ 40 kg/m^2^ have the right to specialized healthcare treatment including bariatric surgery, pharmacotherapy, or lifestyle treatment at rehabilitation centers (18). The choice of treatment requires an assessment of each patient’s needs and possibilities (8). Patients in our study were those who attended lifestyle treatment at Avonova Ringerike Rehabilitation Center (ARRC).

Previous research has shown that rehabilitation centers that offer lifestyle treatment with continuous stays for patients with obesity lead to favorable outcomes (8), including improvements in blood lipid levels, blood pressure, insulin sensitivity, and psychological well-being, regardless of their impact on body fat (19, 20). Negative aspects are that these comprehensive treatments can be resource-intensive and their effectiveness may be impacted by external factors (21). To enhance and sustain the effectiveness of these lifestyle treatments, it is crucial to develop innovative strategies that address both physiological and behavioral challenges while tailoring treatment to individual needs. Moreover, when transitioning from continuous stays at rehabilitation centers to partially digital follow-up at home, continuous evaluation is crucial to optimize treatment and ensure its efficacy in supporting patients (17). Hence, this study aims to compare three health outcomes—BMI, HRQoL, and PA levels—between patients with obesity who attended usual in-person treatment before COVID-19 at the ARRC with those who attended treatment during COVID-19 with reduced physical attendance at the ARRC and increased digital follow-up.

## Methodology

### Study design

A retrospective pre-post design was used for this study.

### Recruitment

**Figure 1:**
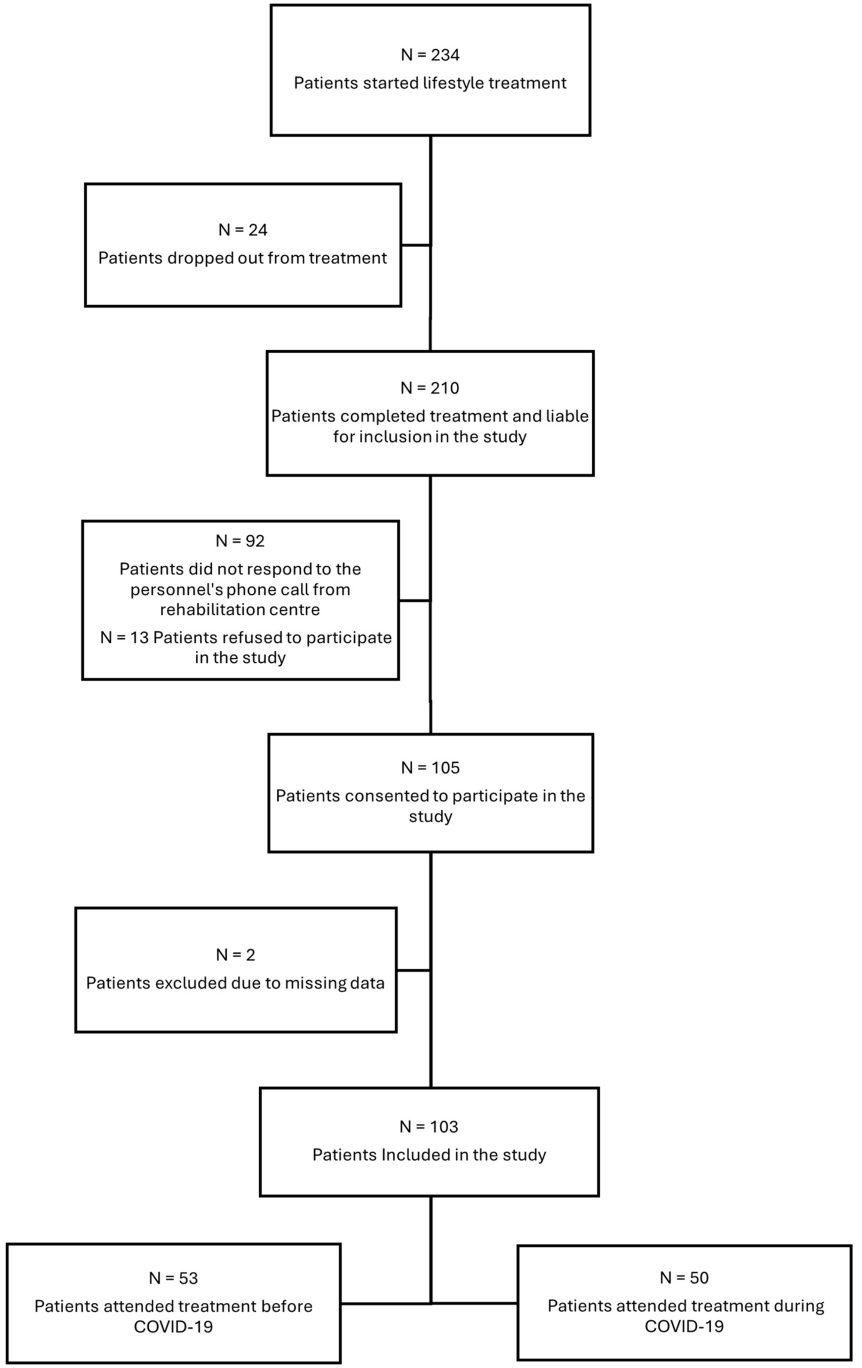
Recruitment of patients from the treatment at ARRC to study participation.

Patients with a BMI ≥ 35 kg/m^2^ with comorbidities, or a BMI ≥ 40 kg/m^2^, were referred from the Center for Morbid Obesity in Tønsberg to the lifestyle treatment at the ARRC. Criteria to attend the lifestyle treatment were patients 18 years or older, motivated to change, and those who had tried municipal or local variants of lifestyle treatment options before (22). Patients’ motivation to change was assessed through clinical interviews during intake, while prior attempts at municipal or local lifestyle treatment were evaluated based on their documented health profiles in the healthcare system and referral information. Altogether, 234 patients started the lifestyle treatment before and during COVID-19, but 24 patients (10%) dropped out from treatment during the main stay without providing a reason. Of the 210 patients who completed treatment and liable for inclusion in the study, personnel from the ARRC contacted them via phone to invite participation in this study. A total of 105 patients expressed interest and provided electronic consent. Due to missing data from two patients, the final sample included in the study were 103 patients, resulting in a 50% participation rate. Of these, 53 patients attended treatment before COVID-19 (just before 2019), and 50 patients attended treatment during COVID-19 (in 2020). The inclusion process for patients in this study is illustrated in Figure 1.

The baseline characteristics of the 103 patients who have been included in the study are demonstrated in Table 1.

**Table 1:** Baseline characteristics of all patients included in the study.

Variables	Patients before COVID-19 (*n* = 53)	Patients during COVID-19 (*n* = 50)	Confidence interval, *p* value (*p* < 0.05)*
Age (SD)	45.3 (± 11.5)	45.5 (± 11.2)	(−4.69, 4.20) *p* < 0.911
Gender (%) Women	35 (66%)	31 (62%)	*p* < 0.673
Body mass index (kg/m^2^; SD)	42.8 (± 6.3)	41.7 (± 5.8)	(−1.26, 3.47) *p* < 0.356
Health-related quality of life visual analog scale score (SD)	48.8 (± 20.5)	43.8 (± 15.9)	(−2.22, 12.17) *p* < 0.174
Physical activity levels (Functional threshold power; SD)	116.0 (± 45.5)	119.1 (± 50.0)	(−22.55, 15.31) *p* < 0.702

Values in Table 1 present that patients were aged 21–71 years, with an average of 45.4 years, 64% were women. Their mean BMI at baseline was 42.3 kg/m^2^ (± 6.1). Differences between before and during COVID-19 groups were assessed using independent *t*-tests for normally distributed continuous variables, Wilcoxon–Mann–Whitney test for non-normally distributed continuous variables, and Chi2-test for categorical variables. Abbreviations: SD; standard deviation, *n*; number. *Significance level.

### Content of the lifestyle treatment program at the ARRC

**Figure 2:**
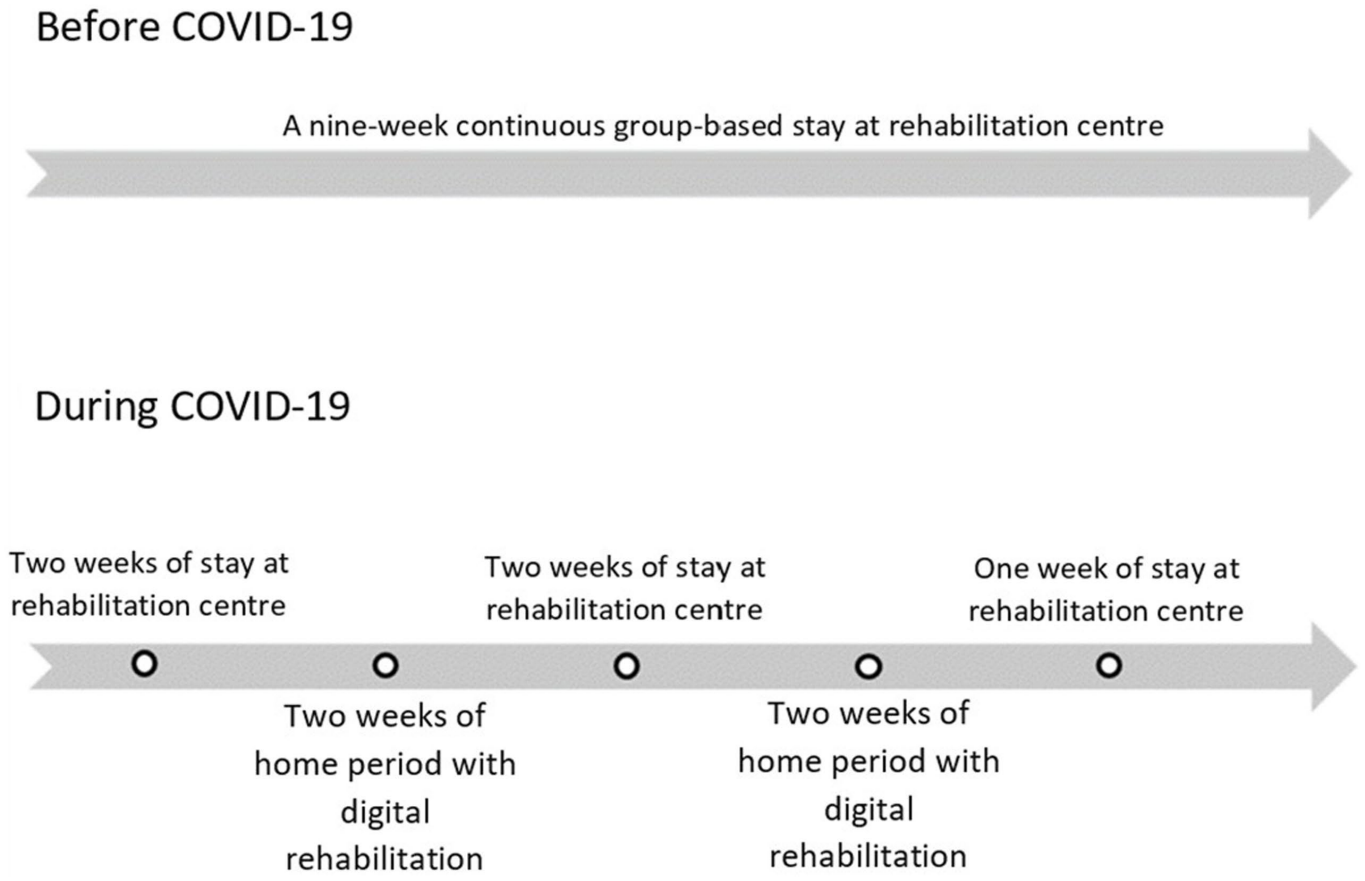
Demonstration and description of the treatment before and during COVID-19.

The lifestyle treatment program at the ARRC was based on recommendations from the Norwegian Directorate of Health (18). A multidisciplinary team consisted of a psychologist, doctor, exercise instructor, clinical nutritionist, physiotherapist, nurse, and coordinator who managed the treatment. Before COVID-19, the program consisted of a nine-week continuous (Monday to Friday; 08:00–15:30) group-based rehabilitation program at the ARRC (see Figure 2). There were three compulsory activities, of which two were physical and one was theoretical. Exercise sessions exceeded 60 min daily and were led by an instructor. Within the week, there were two to three walks, two cycling sessions, and one to three indoor training sessions, with resistance training, a yoga session, and pole walking intervals. Seven nutrition-focused theoretical sessions addressed meal rhythm, portion sizes, and meal planning, emphasizing sustainable weight management. Meals, tailored by nutritionists and adjusted for individual needs, followed Norwegian dietary guidelines (23), with women consuming ~1,600 kcal and men ~1900 kcal daily, distributed across three main meals and three snacks.

Cognitive behavioral therapy, delivered in five sessions by a psychologist, combined behavioral strategies with group work to enhance motivation and promote lasting change. Patients practiced coping strategies and developed planning routines by evaluating weekly tasks related to diet, exercise, and mental health challenges. This approach aimed to foster self-awareness, problem-solving, and long-term lifestyle changes.

### Changes in the lifestyle treatment program due to COVID-19 pandemic

During COVID-19, parts of the continuous group-based rehabilitation program at the ARRC were replaced with digital follow-up from home. The program included 5 weeks at the ARRC (weeks 1, 2, 5, 6, and 9) and 4 weeks at home (weeks 3, 4, 7, and 8; see Figure 2). During the home period, patients were required to adhere to a structured timetable, necessitating a high degree of self-discipline. They were responsible for developing a plan that incorporated both dietary guidelines and physical activity. To support this process, a web-based portal (Teachable.co) provided access to timetables, instructional materials, training videos, practical tips, and other resources. Each Sunday, patients completed planning and evaluation forms to track their progress. Additionally, the interdisciplinary team conducted individualized follow-up phone calls every Monday, Wednesday, and Friday. Beyond these scheduled calls, patients could communicate with the team through a secure web-based platform.

### Data collection

After obtaining consent from patients, data was collected from the web-based journal system Extensor Rehab used at the ARRC. Data was de-identified, and all patients were linked to a six-digit code. The material was then extracted in an Excel file that was transferred to SPSS for further analysis. Sociodemographic variables included in the study were age (years), and sex (women/men), which were obtained from the patient’s records. Anthropometrical variables were height in centimeters which was measured at first consultation by a doctor using the Seca 206 measuring device (0–220 cm). For this measure, patients stood barefoot with heels together against the wall with the entire sole of the foot placed on the floor and the body in a straight position. Weight in kilograms was measured by Marsden M-530 (0–300 kg) where the patient was wearing thin and light sports clothes but no shoes. BMI was calculated based on weight divided by the square of height in meters (kg/m^2^). Physical Activity levels were measured with a Functional Threshold Power (FTP) test carried out in a spinning room on a TOMAHAWK IC7 indoor bike with an instructor. The result is measured by energy per unit of time with measure watt (W) defined as one joule per second (1 J/s) (24). HRQoL was assessed by the European Quality of Life 5 Dimensions 5 Level Version (EQ-5D-5L), a widely used generic tool developed by the European Quality of Life Group (25). It has been shown to have good validity and reliability as a measure of HRQoL (26). EQ-5D-5L is divided into five dimensions: walking, personal care, usual chores, pain/discomfort, and anxiety/depression. In each of these categories, patients gave a rating on a five-point scale, where 1 equal “no problems” and 5 corresponds to “major problems.” The patient’s self-assessed health was also reported on a visual analog scale (VAS score) numbered from 0 to 100, where 100 indicates the best.

### Ethics statement

The Regional Committee for Medical and Health Research Ethics in Central Norway (REK) approved the study (REK reference number 211470) and patients signed a digital informed consent.

### Analysis

Statistical analyses were carried out in Statistical Package for the Social Sciences (IBM SPSS Statistics) version 28. Descriptive data were presented with means (SD) and numbers (%). Repeated student’s t-test was used for the continuous normally distributed variables. For the non-normally distributed continuous variables, the Wilcoxon-Mann–Whitney test were used. The significance level was set at 5% (*p* < 0.05). Multiple imputation in SPSS was used to impute the missing variables. The method estimates the missing values in the dataset based on the remaining variables in the analysis model. When the proportion of missing data is low (≤5%), this method is considered fairly accurate (27).

## Results

A total of 103 patients (*n* = 53 before COVID-19 and *n* = 50 during COVID-19, aged 45.4 years; with 64% women; BMI 42.3 ± 6.1 kg/m^2^) were included in this study (see Table 1). All patients had reduced BMI (*p* < 0.001) after 9 weeks of treatments, with a mean BMI reduction of - 3.5 kg/m^2^ (± 1.62). There was no significant difference in BMI between the patients before and during COVID-19 (*p* = 0.221; see Table 2).

**Table 2:** Changes in health outcomes after treatment for patients before and during COVID-19.

Variables	After treatment, before COVID-19 (*n* = 53)	After treatment, during COVID-19 (*n* = 50)	Confidence interval, *p*-value (*p* < 0.05)*
Body mass index (kg/m^2^; SD)	−3.2 (± 1.18)	−3.7 (± 1.99)	(−0.94, 0.32), *p* = 0.221
Health-related quality of life visual analog scale score (SD)	+ 26.9 (± 16.3)	+ 21.1 (± 14.5)	(0.98, 13.39), *P* = 0.001*
Physical activity Levels (Functional threshold power; Watt; SD)	+ 45.1 (± 26.0)	+ 25.2 (± 38.1)	(−35.7, −7.58), *p* = 0.003*

Values in Table 2 are presented as means (± SD) and significance level (*p*). Changes in health outcomes between before and during COVID-19 groups were assessed using independent *t*-tests for normally distributed continuous variables, Wilcoxon–Mann–Whitney test for non-normally distributed continuous variables. Abbreviations: SD; standard deviation, n; number. *Significance level.

**Figure 3:**
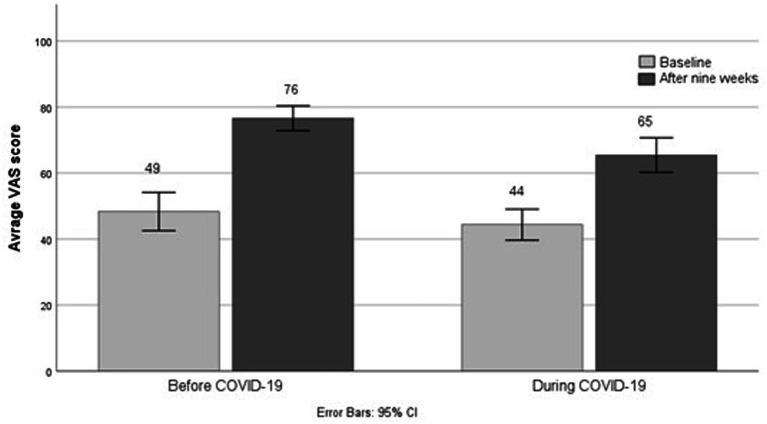
Average VAS score of patients’ self-assessed health for the groups before and during COVID-19.

All patients had improved VAS scores after 9 weeks of treatments (*p* < 0.001). However, there was a difference in HRQoL between the patients before and during COVID-19 groups (*p* = 0.001). Patients before COVID-19 had higher increase in VAS score (+ 26.9, ± 16.3) compared to the patients during COVID-19 (+ 21.1, ± 14.5; see Figure 3). Changes in health outcomes after treatment for both groups are presented in Table 2.

**Figure 4:**
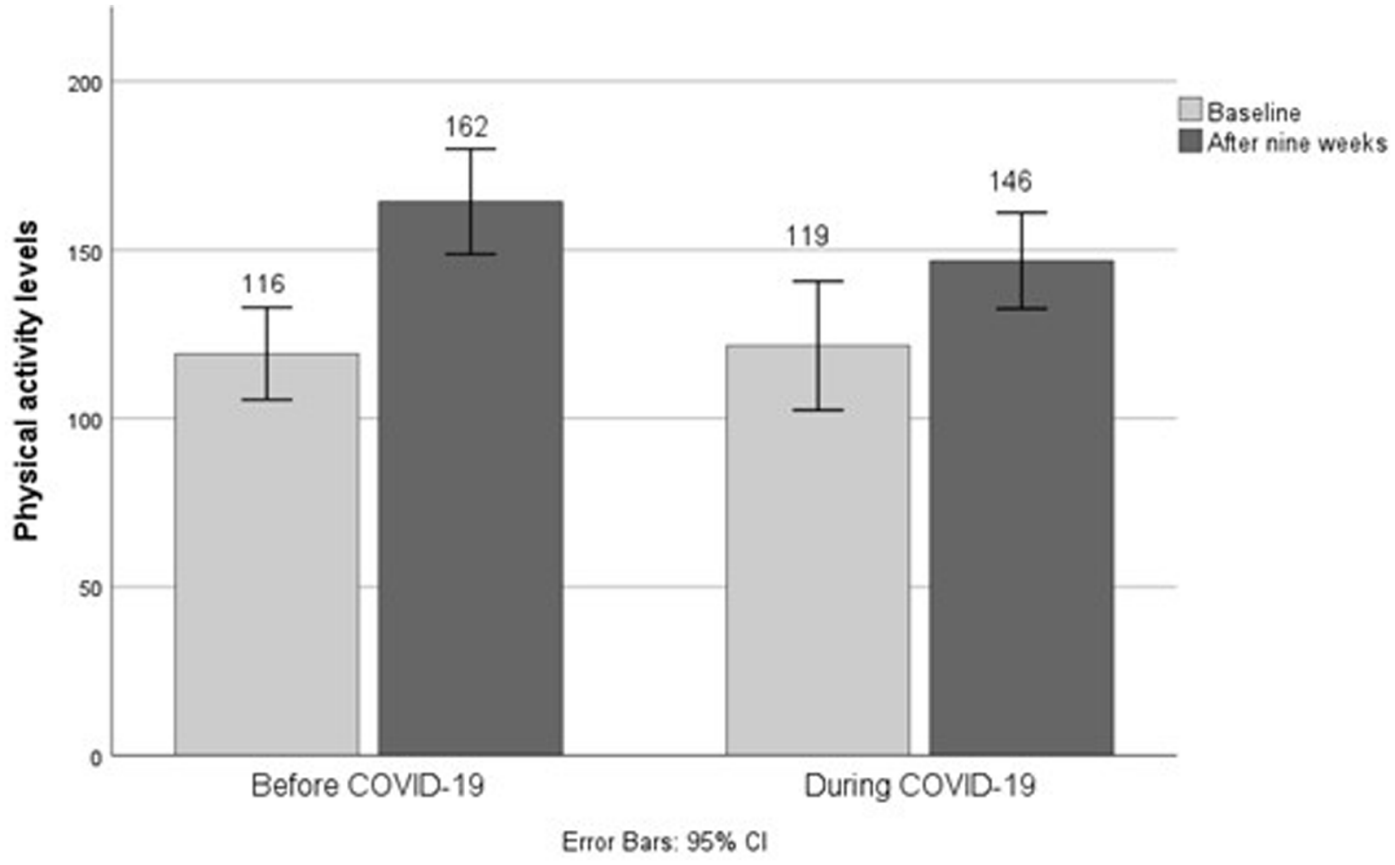
Physical activity levels for the groups before and during COVID-19.

For the PA levels, all patients showed an improvement (*p* < 0.001) with an average of + 35 Watts (± 32.7). There was a difference in PA levels between patients before and during COVID-19 groups (*p* = 0.003; see Table 2), such that patients before COVID-19 had increased PA levels with a mean of + 45.1 Watts (± 26.0) and patients during COVID-19 had increased their PA levels by a mean of + 25.2 Watts (± 38.1; see Figure 4).

## Discussion

This study compared three health outcomes between patients who attended lifestyle treatment before COVID-19 and patients who attended during COVID-19. The main findings show that BMI reduction was similar in both groups. While HRQoL and PA levels increased for both groups after treatment, the before-COVID-19 group reported higher HRQoL and had more improvement in PA levels compared with the during-COVID-19 group.

### Impact of the COVID-19 pandemic on HRQoL in patients with obesity attending lifestyle treatment

Our study found that while both groups experienced improvements in HRQoL, patients who attended treatment before COVID-19 reported higher HRQoL scores compared to those who attended during the COVID-19. This suggests that other factors specific to the pandemic period may have negatively impacted their quality of life. Our findings align with previous research showing that the COVID-19 stay-at-home order was associated with significant mental health challenges among individuals with obesity (11, 12, 28). Research indicates that 73% of patients receiving care from both an obesity medicine clinic and a bariatric surgery practice reported heightened anxiety, 84% experienced increased depression, and 70% faced challenges with weight management during the pandemic (11). Additionally, pandemic-related concerns could have influenced HRQoL. Early in the pandemic, individuals with obesity were believed to have a higher risk of severe COVID-19 outcomes (29), a concern later confirmed by studies showing increased risks of infection and mortality (30).

In addition, disruption in social support could be another possible factor that may have affected HRQoL among the patients during the COVID-19 pandemic. Societal restrictions and recommendations to reduce social contact during the pandemic may have disrupted essential social support from family and friends, especially during home-based treatment phases (31). Social support is a key motivator for successful weight loss and maintenance, with group-based approaches often outperforming solo methods—where individuals undertake the effort independently or with minimal external, structured support—as well as one-to-one methods (32). Humans, as social beings, tend to address challenges more easily within a supportive, secure group environment (33). Group-based conservative treatment, which includes education and group training sessions with like-minded individuals, has been shown to enhance motivation and coping skills (34). At the rehabilitation center, mandatory participation in educational and physical sessions provided structured support and facilitated group interaction, likely contributing to increased motivation and coping capacity among patients. In contrast, home-based components of the treatment during the pandemic required greater self-discipline on the patients’ side, potentially lowering HRQoL outcomes. Moreover, patients who did not adhere to PA or dietary plans may have felt less capable of coping, further reducing HRQoL. Future studies should explore lifestyle treatments that integrate mental health support and social connections, particularly during periods of social restrictions.

### The impact of COVID-19 pandemic on PA levels in patients with obesity attending lifestyle treatment

Both groups in our study showed improvements in PA levels, measured by functional threshold power. This aligns with previous research demonstrating increased physical fitness among patients with obesity attending lifestyle treatment (35). The COVID-19 lockdowns, as noted in other studies (11, 12, 36), disrupted exercise routines, leading to a decline in PA levels, increased sedentary time, and more time spent in bed during weekdays. This may explain the disparity in PA levels between our study groups before and during pandemic. A study examining the effects of COVID-19 stay-at-home orders on weight-related behaviors in patients with obesity found that the pandemic had a significant impact on these behaviors. Patients experienced a reduction in intensity and duration of PA, along with difficulties in achieving weight loss goals, regardless of infection (11). In another study exploring the challenges of weight management and improving lifestyle treatment during COVID-19, participants reported that stress and anxiety caused by stay-at-home orders further hindered adherence to weight management programs (15). An online survey comparing PA levels before and during COVID-19 pandemic found that people with obesity were disproportionately affected, experiencing a decline in PA, a shift toward lower-intensity activities, and increased sedentary behavior, especially on weekdays (13). Secondly, the program structure likely influenced the disparity in PA levels between groups in our study. The before-pandemic group attended all mandatory classes and daily supervised exercise sessions, promoting better adherence and improvement in PA. In contrast, the COVID-19 program’s at-home component relied on patient self-discipline, making it harder to maintain consistent exercise. Such challenges are supported by research indicating difficulties with self-directed exercise, particularly for individuals with weight management goals (37). Furthermore, existing knowledge aligns with the understanding that, while exercise may not be the most effective initial weight loss strategy, consistent exercise is a crucial factor for long-term weight maintenance success and cardiovascular health (38). Altogether, the structured and supervised environment of the before-COVID-19 program likely facilitated the development of sustainable exercise habits, contributing to the observed greater improvement in PA levels.

### The interplay between PA levels and HRQoL in lifestyle treatment

Given the observed differences in PA levels, it is important to consider how these differences may relate to HRQoL among individuals in our study. Increased isolation during home confinement has been unfavorable for patients with obesity, contributing to reduced PA and further decline in HRQoL (39). Physical fitness—often as a proxy for PA—may reduce obesity-related mortality and improve both physical and mental aspects of quality of life, even if its link to weight loss is less clear (14, 20, 28). In our study, patients who attended treatment before COVID-19 with higher PA levels demonstrated greater HRQoL scores compared to those who showed lower PA improvements during the pandemic. This aligns with the existing literature (40), which suggests that even a slight improvement in PA can enhance HRQoL in individuals with obesity. However, the relationship between PA and HRQoL is complex (14, 41). While one study supports our findings by demonstrating an association between PA and HRQoL (14), another suggests that the impact of PA varies across different dimensions of HRQoL. For instance, one study revealed that PA primarily improves the physical aspects of HRQoL but has limited effects on the mental aspect of it, particularly in individuals undergoing obesity treatment (42). Conversely, one study (41) reported no significant improvement in HRQoL following structured PA interventions, highlighting that factors beyond PA such as psychological or lifestyle factors, may play a crucial role in determining HRQoL outcomes. Moreover, one study has found that lifestyle interventions lead to notable improvements in quality of life (43).

Both our findings and existing literature (14, 41), underscore the intricate relationship between PA, mental health, and HRQoL in people with obesity, particularly within the context of the COVID-19 pandemic. The pandemic exacerbated mental health challenges, reduced PA levels, and negatively affected HRQoL (11, 12), reinforcing the need for comprehensive, socially supportive lifestyle treatments. However, as this study was a natural experiment without randomization, we cannot fully exclude the possibility that other external factors contributed to the observed differences. Future weight management strategies should prioritize structured, accessible, and guided interventions that address self-discipline challenges and promote long-term health maintenance.

## Strengths and limitations

This study’s retrospective pre-post design presents distinct strengths alongside notable limitations. A key strength of this study design is its ability to account for patient dropouts, as outcomes and follow-up data were already available at the time of analysis, which is a common challenge in long-term follow-up studies (44). Although the study sample includes more women than men, reflecting typical referral patterns for lifestyle treatments (45), it is the relatively small sample size, rather than the gender imbalance itself, that may have limited the ability to detect sex-related differences. As the sample closely represents the demographics of patients referred for lifestyle treatment, the results may be generalizable to this population group. Furthermore, the requirement for active consent may have introduced selection bias and potentially overrepresented patients who were more satisfied with their outcomes. Another strength lies in the choice of measurement tools. The EQ-5D-5L is broadly applicable and widely used in assessing HRQoL across various conditions (25), yet it lacks obesity-specific sensitivity compared to tools like Impact of Weight on Quality of Life-Lite Clinical Trials (IWQOL-Lite CT) (9). The IWQOL-Lite questionnaire is license-based and the implementation of such a measurement instrument at the rehabilitation center will probably be a cost issue. Similarly, although maximal oxygen uptake (VO_2_ max) testing is considered the gold standard for cardiorespiratory fitness assessment (46), the study was chosen for cycling as a practical alternative. Cycling is particularly beneficial for patients with obesity due to its low joint impact, enhancing accessibility, especially given the mobility limitations in this population (18). Lastly, the study’s strength in terms of data completeness should be noted: missing data were below 5% for PA levels at baseline and HRQoL at the end of the treatments. This low level of missing data supports the reliability of findings when using multiple imputations (27). However, it is important to recognize that the retrospective design is also vulnerable to parallel phenomena, meaning that other factors unrelated to the treatment may have influenced the results. Furthermore, causal relationships cannot be confirmed in a retrospective study, highlighting a significant limitation in interpreting the findings (44).

## Conclusion

This study demonstrates that structured lifestyle treatment supports improvements in BMI, HRQoL, and PA levels, even when delivered with reduced in-person contact. Although differences were observed between treatment periods, the findings underscore the potential of structured interventions to support individuals with obesity. Based on this study’s findings, flexible and hybrid models, including digital components, can be effectively implemented in clinical obesity treatment to support BMI reduction. However, treatment should emphasize mental health support and actively promote social connection to enhance overall outcome. Further research is needed to investigate how different components and delivery modes of lifestyle treatments may influence health outcomes and to explore their long-term effects.

## Data Availability

Publicly available datasets were analyzed in this study. This data can be found at: The datasets supporting the conclusion of this article are available in the Center for Obesity Research (ObeCe), St. Olavs Hospital, Trondheim, Norway.
